# Long-term outcomes following bilateral pilon fractures: a report of three cases

**DOI:** 10.1093/jscr/rjaf826

**Published:** 2025-10-17

**Authors:** Nicholas Frappa, Danil Chernov, Morgan Dillon, Matthew G Alben, Joshua Slowinski, Samuel I Fuller, Alexander Kovacs, Ryan Riley, Susan Daoust

**Affiliations:** Jacobs School of Medicine and Biomedical Sciences, 955 Main St. Buffalo, NY 14203, United States; Jacobs School of Medicine and Biomedical Sciences, 955 Main St. Buffalo, NY 14203, United States; Jacobs School of Medicine and Biomedical Sciences, 955 Main St. Buffalo, NY 14203, United States; University at Buffalo Department of Orthopaedics and Sports Medicine, 462 Grider St. Buffalo, NY 14215, United States; University at Buffalo Department of Orthopaedics and Sports Medicine, 462 Grider St. Buffalo, NY 14215, United States; University at Buffalo Department of Orthopaedics and Sports Medicine, 462 Grider St. Buffalo, NY 14215, United States; University at Buffalo Department of Orthopaedics and Sports Medicine, 462 Grider St. Buffalo, NY 14215, United States; University at Buffalo Department of Orthopaedics and Sports Medicine, 462 Grider St. Buffalo, NY 14215, United States; University at Buffalo Department of Orthopaedics and Sports Medicine, 462 Grider St. Buffalo, NY 14215, United States

**Keywords:** bilateral ankle fractures, pilon fracture, nonunion, bilateral pilon fracture

## Abstract

We present three male patients who sustained bilateral pilon fractures following high-energy trauma, including falls and motor vehicle collisions. All patients had comorbidities such as obesity, tobacco use, and cardiovascular, or psychiatric conditions. Each underwent staged open reduction and internal fixation using contemporary techniques tailored to fracture pattern and soft tissue status. Over a minimum follow-up of 2 years, two patients experienced significant late complications, including distal tibial nonunion and symptomatic post-traumatic arthritis. These required revision procedures, such as iliac crest bone grafting, tibiotalar arthrodesis, and subtalar fusion, >1 year after index surgery. This is the first case series to report long-term outcomes following bilateral pilon fractures. Our findings underscore the importance of extended postoperative surveillance and suggest that bilateral injuries may portend a more complex recovery. Further research is warranted to guide prognosis, optimize treatment, and improve counseling for patients with complex bilateral lower extremity trauma.

## Introduction

Pilon fractures are high-energy injuries of the distal tibia that result in substantial comminution of the articular surface and are frequently accompanied by significant soft tissue damage, making them complex to manage and prone to complications [1–4]. The management of pilon fractures commonly involves open reduction and internal fixation (ORIF), often following external fixation in a staged fashion to mitigate risks of infection and wound complications [2, 5]. Despite advancements in surgical strategies, outcomes remain unpredictable, with a high incidence of long-term complications such as nonunion, chronic pain, post-traumatic arthritis, and functional limitations [2–4].

While extensive literature exists on isolated pilon fractures, bilateral cases are exceedingly rare and present unique clinical challenges [6, 7]. To our knowledge, only two case reports have described bilateral pilon fractures, with relatively short follow-up periods of 6 and 10 months, respectively [6, 7]. The lack of long-term data leaves a gap in understanding the full recovery trajectory and complications of such injuries.

This case series presents three patients with bilateral pilon fractures treated surgically, each with a follow-up period extending beyond 2 years. We aim to provide a comprehensive understanding of the long-term outcomes of bilateral pilon fractures, offering new insights into the recovery and challenges faced by these patients. All patients in this case series were informed that data concerning their cases would be submitted for publication, and they provided consent.

## Case reports

### Patient 1

#### Case presentation

**Figure 1:**
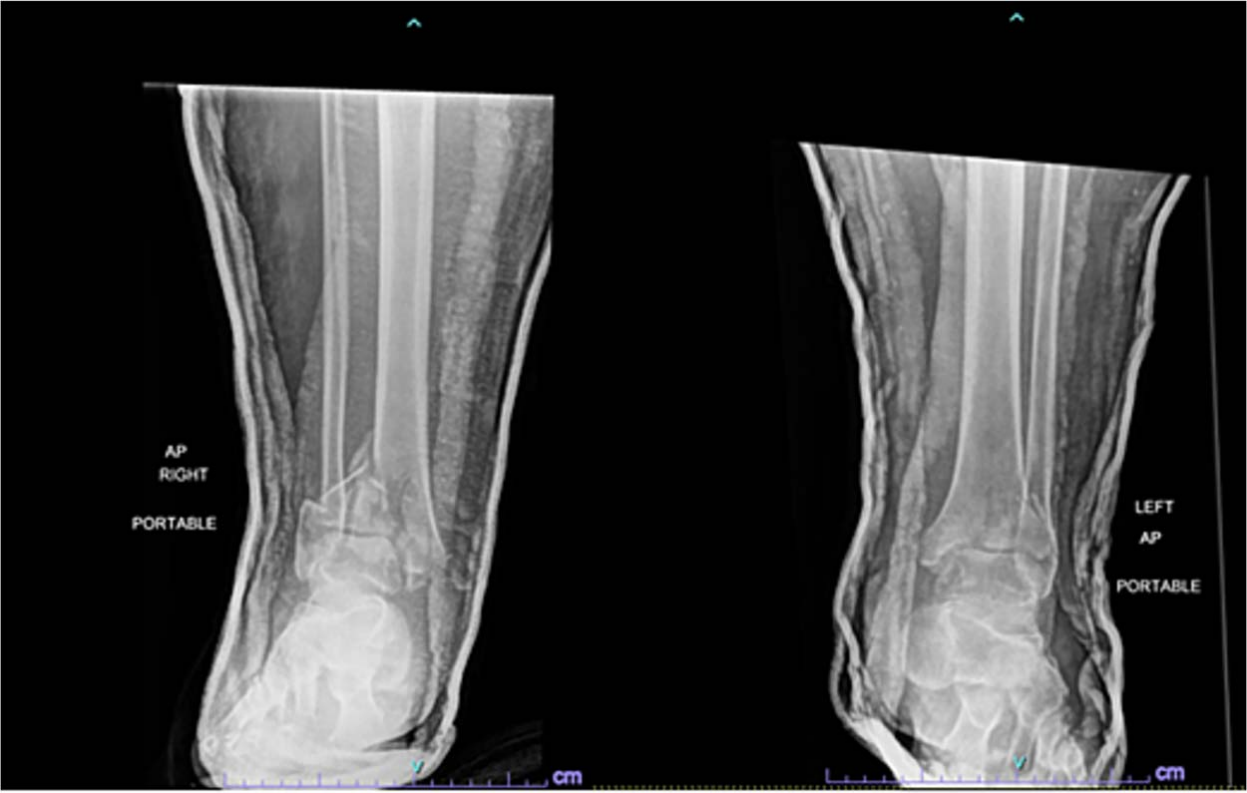
Anterior-to-posterior (AP) radiographs of patient 1 obtained 10 days after injury, upon transfer to our facility, demonstrating bilateral pilon fractures.

A 57-year-old male with a body mass index (BMI) of 48.7 kg/m^2^ and multiple comorbidities, including asthma, coronary artery disease, hypertension, hyperlipidemia, ischemic cardiomyopathy, obstructive sleep apnea, and hypothyroidism, following a motor vehicle collision when he lost control of his vehicle due to a coughing spell. His injuries included bilateral pilon fractures (Fig. 1), a T2 superior endplate fracture, and bilateral pulmonary contusions. He initially presented to a regional hospital and was transferred to our facility 10 days after the injury.

Surgical treatment occurred 2 days later. The left leg was treated via an anterior approach, with excision of several joint fragments and fixation using anterolateral and medial plates. The right leg underwent fixation with a distal fibular plate and placement of a circular ring fixator, which was removed 2 months later. The patient was initially wheelchair dependent because of non-weightbearing (NWB) on the left and toe-touch weightbearing (TTWB) on the right. After removal of the fixator, he transitioned to crutches with weightbearing as tolerated (WBAT) on the left and continued NWB on the right. He was compliant with these restrictions.

#### Outcome

**Figure 2:**
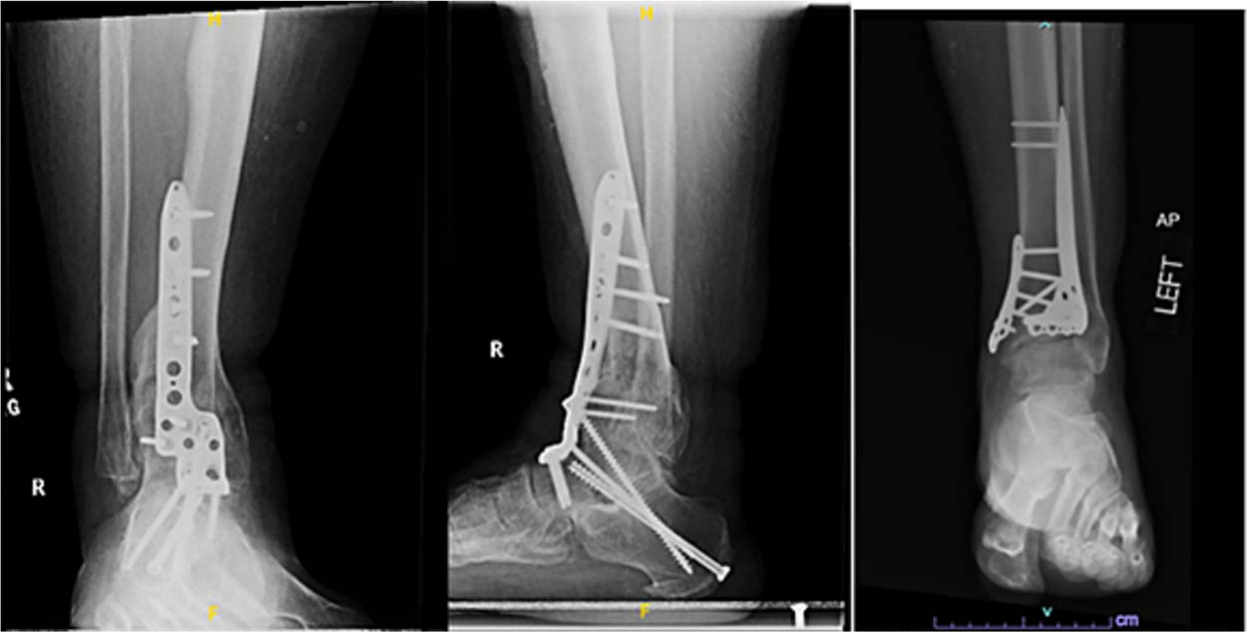
Radiographs of patient 1 obtained 3 years after the index procedure, including AP view of the left ankle and lateral and oblique views of the right ankle following subtalar fusion.

One year after the initial surgery, the left leg healed without any complications. However, the right leg developed nonunion through the distal tibia, along with posttraumatic arthritis. As a result, the patient underwent a right ankle fusion. Additionally, 2.5 years after the index surgery (1.5 years after the ankle fusion), the patient required removal of the right lateral fibular plate due to painful hardware. Six months later (3 years post-index surgery), the patient then underwent subtalar fusion due to post-traumatic arthropathy and ongoing pain (Fig. 2).

### Patient 2

#### Case presentation

**Figure 3:**
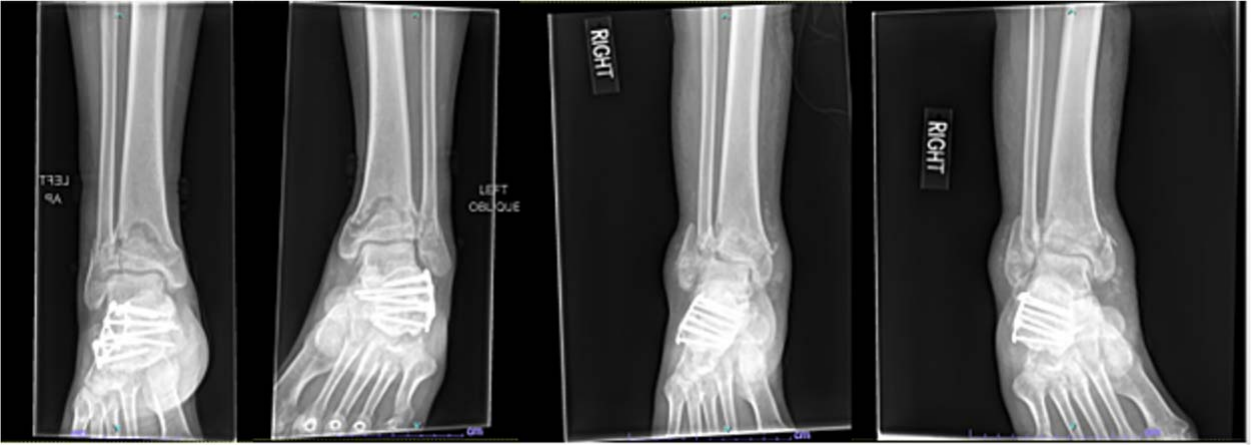
AP and oblique radiographs of patient 2 obtained after a fall, demonstrating bilateral pilon fractures and retained hardware from prior bilateral calcaneal ORIF.

A 38-year-old male with a BMI of 27.5 kg/m^2^ and a history of alcohol use disorder, paranoid schizophrenia, smoking, and prior bilateral calcaneal fractures treated with ORIF 4 years earlier sustained bilateral pilon fractures after jumping from a third-story window (Fig. 3). He presented to the hospital 5 days after injury.

Initial surgery occurred 3 days after presenting to the hospital and involved definitive ORIF of the left leg using a distal tibial locking plate and a distal fibular locking plate. At the time of the initial surgery, there were significant fracture blisters that prevented definitive fixation of the right leg; thus, an external fixator was applied to the right leg. One week later, ORIF of the right leg was performed with distal tibial and fibular locking plates. The patient was made NWB bilaterally and required a wheelchair for mobility during the first 12 weeks. Although he was instructed to use crutches once partial weightbearing (PWB) was permitted, he demonstrated poor adherence to these restrictions.

#### Outcome

**Figure 4:**
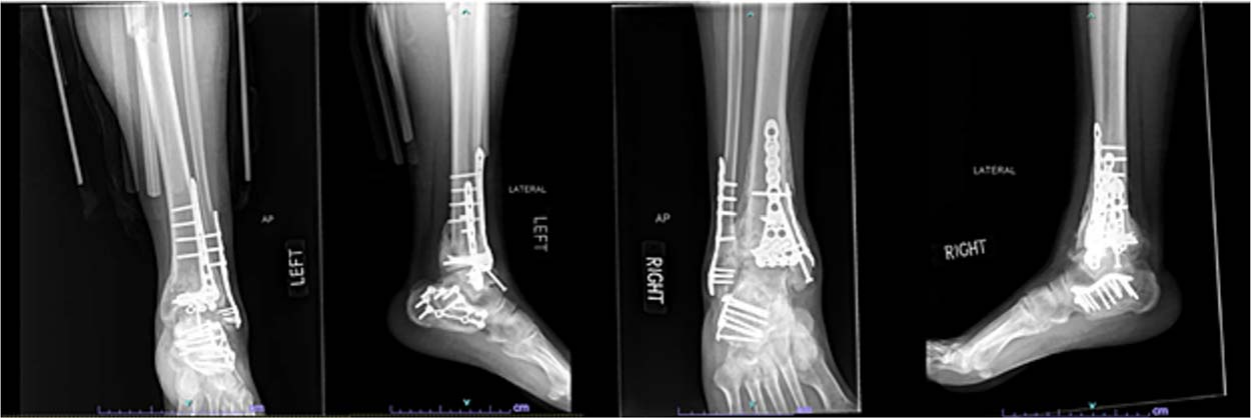
Two-year follow-up AP and lateral radiographs of both ankles in patient 2 showing maintained alignment and hardware position.

Despite the patient’s noncompliance, he went on to heal uneventfully for the next 2 years until the patient fell from a height of 15 feet, resulting in a left bicondylar tibial plateau fracture and fracture of the left tibial shaft. These injuries required ORIF of his left tibia. Despite the new injury, the hardware from the pilon fractures was in proper alignment (Fig. 4).

### Patient 3

#### Case presentation

**Figure 5:**
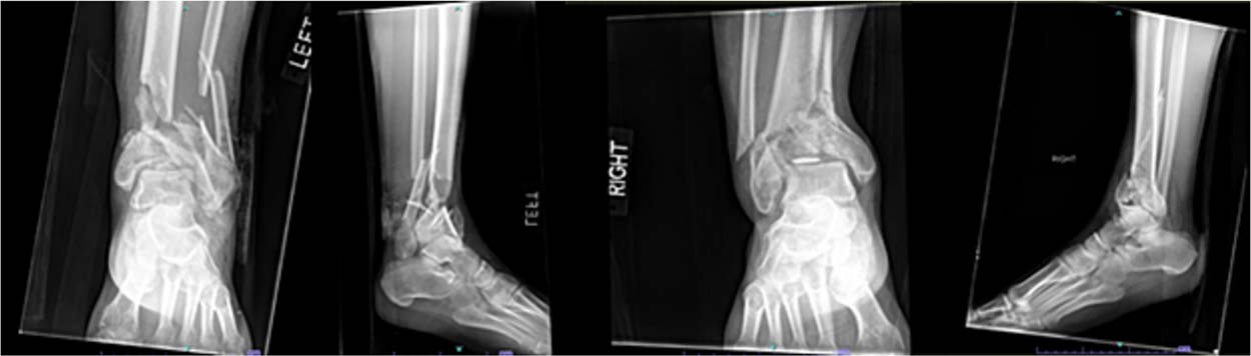
Initial AP and lateral radiographs in patient 3 demonstrating bilateral open pilon fractures with severe comminution of the left ankle.

A 42-year-old male with a BMI of 33.7 kg/m^2^ and history of asthma, marijuana use, and vaping use presented after a 30-foot fall from a ladder while at work. In addition to bilateral open pilon fractures (Fig. 5), he sustained nondisplaced fractures of the L2 and L3 transverse processes and a minimally displaced posterior fracture of the right 12th rib. He underwent initial surgery the same day.

Initial surgery involved lateral incisions on both legs with placement of lateral locking plates. On the left, a separate anterior incision through the extensor retinaculum was also performed to reduce a displaced plafond fragment. Bilateral spanning external fixators were then applied. Three days later, the fixators were removed, and definitive fixation was performed bilaterally using anteromedial approaches with placement of medial and anterolateral distal tibial locking plates. The patient was initially NWB bilaterally and relied on a wheelchair for mobility. At 12 weeks, partial weightbearing was permitted bilaterally, allowing transition to crutches. He complied with rehabilitation instructions and participated in physical therapy.

#### Outcome

**Figure 6:**
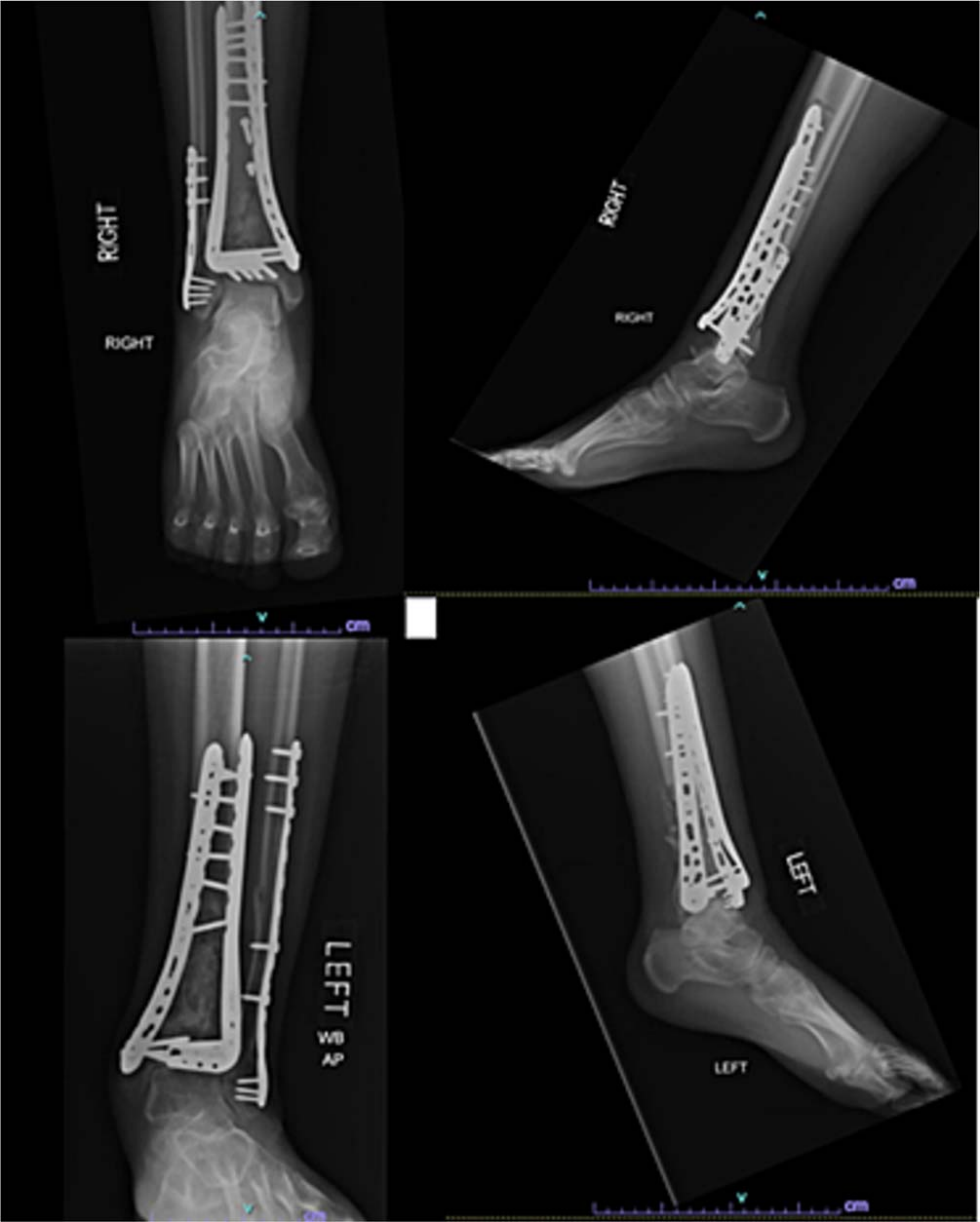
AP and lateral radiographs of patient 3 taken 3 months after posterior iliac crest bone grafting (7 months after index procedure).

**Figure 7:**
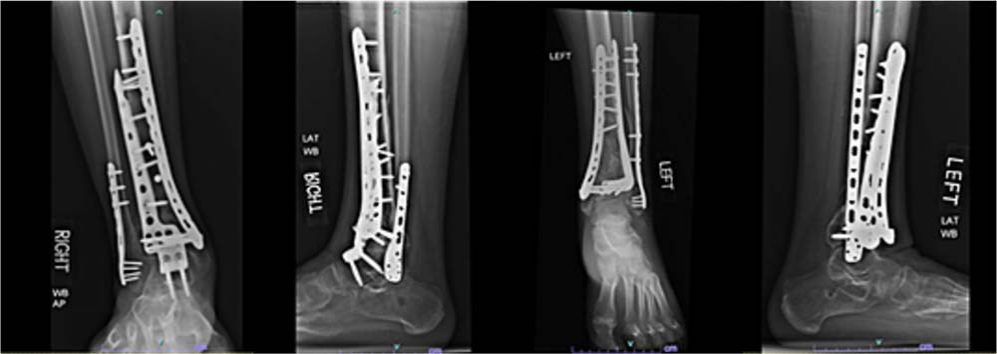
AP and lateral radiographs of patient 3 obtained 1 year and 9 months after the index procedure, demonstrating right ankle arthrodesis and a healed left ankle.

Seven months after the initial surgery, the patient continued to experience pain in the left leg, and X-rays revealed nonunion of the distal tibia. This necessitated revision surgery using a posterior iliac crest bone graft to promote healing (Fig. 6). Additionally, 1.5 years after the index surgery, the patient developed ongoing pain from post-traumatic arthritis and subsequently required right tibiotalar arthrodesis to address his persistent symptoms (Fig. 7).

The patients’ injury mechanism, treatment, comorbidities, and outcome are summarized in Table 1.

## Discussion

Bilateral pilon fractures are rare injuries resulting from high-energy axial loading mechanisms, often associated with significant comminution, joint disruption, and soft tissue injury [1–3]. Although the surgical principles guiding treatment are well established, outcomes remain inconsistent, and the risk of poor functional outcomes remains elevated [8–10]. The existing literature on bilateral pilon fractures is limited; only two prior case reports, by Pande and Meisheri [6] and Tiedeken *et al.* [7], described bilateral cases, both with follow-up periods limited to 6 and 10 months, respectively. Our series adds novel insight with extended follow-up beyond 2 years and highlights the onset of late complications. Patient 1 developed nonunion of the left tibia and posttraumatic arthritis, requiring revision surgery, tibiotalar fusion, and ultimately a subtalar fusion 3 years later. The right leg was significantly shortened and fixed in situ after more than 10 days of malreduction prior to definitive fixation. This prolonged malalignment may have contributed to the development of nonunion and progressive arthropathy. Patient 3 experienced nonunion of the left tibia, necessitating bone grafting within 1 year, he later required tibiotalar arthrodesis of the right ankle due to ongoing pain at 1.5 years post-index procedure. These late complications underscore the importance of continued monitoring to detect and address issues such as nonunion and posttraumatic arthritis, which can develop long after the initial surgery.

Smoking has been consistently linked to impaired fracture healing, increased wound complications, and worse functional outcomes [11]. In our case series, Patients 1 and 2 had a history of smoking. Interestingly, Patient 1 experienced the most prolonged recovery and highest number of complications, while Patient 2 did not experience any complications to date of the most recent follow-up. This contrast highlights the multifactorial nature of healing, where comorbidities, injury severity, and other host factors likely interact. There is also mixed evidence regarding the role of obesity in pilon fracture outcomes. Wheelwright et al [12] found that lower BMI was associated with improved performance, and van der Vliet et al [11] similarly demonstrated that higher BMI correlated with poorer scores on the Foot and Ankle Ability Measure. In contrast, Çeçen *et al.* [13] concluded that BMI did not affect overall clinical or radiographic outcomes except for a higher risk of superficial infection. In our series, Patients 1 and 3, both classified as obese (BMI > 30 kg/m^2^), experienced complications. While this is only a case study with three total patients, this is in line with prior findings [11, 12] suggesting that obesity may predispose patients to worse functional outcomes and delayed healing following pilon fracture fixation.

Another important consideration is whether bilateral pilon fractures inherently carry a greater risk of complications compared to unilateral injuries. While the complications of unilateral pilon fractures are well-documented [2–4], it remains unclear whether bilateral injuries confer a greater risk of adverse outcomes, as they likely are secondary to a higher energy mechanism of injury. The additive burden of bilateral articular trauma and the challenges of non-weight bearing on both limbs may intuitively suggest a more difficult recovery. However, no comparative studies to date have clearly established whether bilaterality is an independent risk factor for poor outcomes. This gap in the literature merits further investigation to guide prognosis and optimize patient counseling.

This case series underscores the importance of long-term follow-up in patients with bilateral pilon fractures. Complications such as nonunion and post-traumatic arthritis may emerge well beyond the first postoperative year. Ongoing monitoring allows for timely interventions and may ultimately improve functional outcomes. Larger studies comparing bilateral and unilateral cases are needed to clarify prognostic implications and to refine evidence-based treatment strategies for this rare and challenging injury pattern.
